# The Multiferroic, Magnetic Exchange Bias Effect, and Photodetection Multifunction Characteristics in MnSe/Ga_0.6_Fe_1.4_O_3_ Heterostructure

**DOI:** 10.3390/ma18030586

**Published:** 2025-01-27

**Authors:** Ye Zhao, Ruilong Yang, Ke Yang, Jiarui Dou, Jinzhong Guo, Xiaoting Yang, Guowei Zhou, Xiaohong Xu

**Affiliations:** 1 School of Chemistry and Materials Science of Shanxi Normal University & Key Laboratory of Magnetic Molecules and Magnetic Information Materials, Ministry of Education, Taiyuan 030006, China; zhaoye8901@163.com (Y.Z.); yangruilong@sxnu.edu.cn (R.Y.); 18835736484@163.com (K.Y.); doujr98@163.com (J.D.); 17835095816@163.com (J.G.); matersci2016@163.com (X.Y.); 2 Research Institute of Materials Science of Shanxi Normal University, Taiyuan 030006, China

**Keywords:** multifunction characteristics, multiferroic, magnetic exchange bias effect, photodetection, MnSe/GFO heterostructure

## Abstract

Artificial heterostructures are typically created by layering distinct materials, thereby giving rise to unique characteristics different from their individual components. Herein, two-dimensional α-MnSe nanosheets with a non-layered structure were fabricated on Ga_0.6_Fe_1.4_O_3_ (GFO) films. The superior crystalline properties of MnSe/GFO heterostructures were confirmed through structural and morphological analyses. The remanent polarization is around 1.5 μC/cm^2^ and the leakage current density can reach 2 × 10^−3^ A/cm^2^ under 4 V. In addition, the piezo-response force microscopy amplitude and phase images further supported the ferroelectric property. The significant improvement of coercive field and saturated magnetization, along with the antiparallel signals of Mn and Fe ions observed through synchrotron X-ray analyses, suggest the presence of magnetic interaction within the MnSe/GFO heterostructure. Finally, the excellent photodetector with a photo detectivity of 6.3 × 10^8^ Jones and a photoresponsivity of 2.8 × 10^−3^ A·W^−1^ was obtained under 532 nm in the MnSe/GFO heterostructure. The characteristics of this heterostructure, which include multiferroic, magnetic exchange bias effect, and photodetection capabilities, are highly beneficial for multifunctional devices.

## 1. Introduction

Artificial heterostructures, formed by stacking similar materials, frequently display unique properties that differ from those of their individual constituents. Traditional heterostructures, made up of materials belonging to the same crystallographic family, have long been preferred because they can maintain high crystalline quality with minimal deformities [1,2,3]. However, recent efforts have shifted towards creating heterostructures that merge different material families. These include traditional semiconductor materials and transition metal oxides containing perovskite structure along with the more recent development of 2D materials characterized by layered/non-layered morphologies [4,5,6]. Such heterostructures hold promise for enabling multi-purpose functional systems. For example, highly interdependent electronic systems exhibit a variety of physical phenomena such as superconductivity, ferroelectricity, and ferromagnetism. This is due to the interplay of multiple degrees of freedom, such as lattice, charge, orbital, and spin, leading to complex phase diagrams [7,8,9]. The sensitivity of these substances to changes in parameters positions them as potential candidates for advanced electronic technologies. Nonetheless, several of these parameters are intrinsic to the material characteristics set during synthesis and are hard to manage in real-world settings. Extra functionalities can be achieved by post-growth modifications or by interfacing coupled oxides with various functional materials. Yet most of these oxides are not sensitive to light [10,11]. In contrast, 2D materials such as transition metal dichalcogenides and graphenes, which have either layered or non-layered structures, possess remarkable electronic and optical properties. Because of the weak van der Waals interactions, they can be detached from their growth substrates and transferred to other platforms [12,13]. This makes them highly useful for the integration of heterogeneous systems. Thus, studying the transfer of 2D substances with excellent photoelectric effects onto transition metal oxides with superior magnetoelectric characteristics is of great value for the development of advanced multifunctional technologies.

Manganese selenide (MnSe) in its rock salt (α-phase) structure stands out due to its exceptional photoelectric behavior and diverse crystalline structures [14]. Duan and colleagues noted that α-MnSe, a p-type semiconductor with a wide bandgap and antiferromagnetic characteristics, holds great promise for applications in devices with magnetically induced optical and optoelectronic properties [15]. Zhai and co-workers employed spatially confined chemical vapor deposition (CVD) to synthesize thickness-controlled 2D α-MnSe, investigating its optical and optoelectronic properties to explore the impact of spin configurations on magnetic phase transitions [16]. Currently, the notable p-type semiconductor traits of α-MnSe have been harnessed in optoelectronic tools, and its subtle magnetic properties have also been recently observed in this study. Conversely, multiferroic materials, which exhibit multiple ferroic orders concurrently, including self-induced electric polarization and magnetization within a single phase, have garnered increased interest due to their captivating physics and innovative technological potentials [17,18,19]. Particularly when these materials demonstrate magnetoelectric coupling effects, they offer a promising path for the creation of novel devices with new functionalities, including the manipulation of magnetic properties through electrical fields and vice versa. Gallium ferrite (Ga_0.6_Fe_1.4_O_3_), recognized as a monophasic multiferroic substance, exhibits both ferrimagnetic and ferroelectric properties, with the ability to adjust the magnetic transition at room temperature [20,21]. This versatility is attributed to the pivotal role played by Fe^3+^ ions, influencing not only the magnetic properties but also contributing significantly to the ferroelectric characteristics. Consequently, Ga_0.6_Fe_1.4_O_3_ emerges as a promising candidate among multiferroic materials.

In this study, we constructed a multifunctional heterostructure comprising non-layered α-MnSe and multiferroic Ga_0.6_Fe_1.4_O_3_ (GFO). The superior crystalline performance of MnSe/GFO heterostructures was confirmed through X-ray diffraction (XRD), Raman spectroscopy, and scanning electron microscopy (SEM). Reversals in amplitude and phase observed in piezo-response force microscopy (PFM) indicate polarization switching. Compared to single materials, the MnSe/GFO heterostructure exhibits a significant increase in saturated magnetization and coercive field, indicating the presence of a magnetic exchange bias effect, as evidenced in field-cooling loops. X-ray photoelectron spectroscopy (XPS) and synchrotron X-ray techniques further elucidate the mechanisms of magnetic coupling. Additionally, the MnSe/GFO heterostructure demonstrates exceptional photodetection performance, with a photoresponsivity and detectivity of 2.8 × 10^−3^ A/W and 6.3 × 10^8^ Jones, respectively, at a luminescence wavelength of 532 nm. This research presents a model for developing multifunctional heterostructures by integrating diverse materials.

## 2. Experimental Section

Ultrathin MnSe nanosheets were synthesized via low-pressure CVD in a quartz tube (2-inch) within a two-temperature zone furnace. Selenium (Se, powder, 99.999%, Alfa) was placed upstream and maintained at 370 °C (first heating zone). Manganese(II) chloride (MnCl_2_, powder, 99.9%, Alfa) was positioned in the middle of the furnace at 640 °C. A mica substrate was placed downstream, approximately 4.5 cm from the MnCl_2_ powder, facing downward. Ga_0.6_Fe_1.4_O_3_ (GFO) films, with thicknesses of 45 nm and 60 nm, were grown on (111)-oriented SrTiO_3_ and Nb(0.7%)-SrTiO_3_ substrates via a pulsed-laser deposition (PLD) system under an oxygen atmosphere maintained at a pressure of 100 mTorr. Throughout deposition, the substrates were maintained at 750 °C. A KrF excimer laser used had a wavelength of 248 nm and a fluence of 2.0 J/cm^2^. Non-layered α-MnSe nanosheets were fabricated on f-mica substrate through low-pressure CVD employing high-purity Se and MnCl_2_ as source materials [22]. GFO films of varying thicknesses were deposited on (111)-oriented SrTiO_3_ substrates via PLD. Subsequently, the MnSe nanosheet was first transferred onto polydimethylsiloxane (PDMS) substrate through a wedging transfer technique, followed by a transfer onto the GFO films via a mechanical transfer platform [23].

The MnSe/GFO heterostructure was analyzed using a confocal Raman system equipped with a 532 nm laser, as well as techniques such as X-ray diffraction (XRD), X-ray photoelectron spectroscopy (XPS), and scanning electron microscopy (SEM) combined with energy-dispersive X-ray spectroscopy (EDS). Magnetic characteristics were assessed with a vibrating sample magnetometer within a physical property measurement system, with hysteresis curves derived after eliminating the diamagnetic background. X-ray absorption spectroscopy (XAS) and XMCD measurements were conducted at the National Synchrotron Radiation Laboratory. Macroscopic ferroelectric properties were evaluated using a Radiant Precision Multiferroic II instrument after depositing gold top electrodes (500 μm diameter) via masked thermal evaporation. Furthermore, local ferroelectric (piezoelectric) and magnetic attributes were examined using a piezo-response force microscopy (PFM) module in an atomic force microscope. PFM imaging initially scanned at −8 V outside a designated area (2 × 2 μm^2^), followed by scanning at +8 V within a smaller area (1 × 1 μm^2^). For fabricating devices with two-probe electrodes, laser writing was utilized to deposit Cr/Au (10/80 nm) onto the MnSe sample. The electrical and optoelectronic properties were characterized using a four-probe setup with a Keithley 4200 semiconductor analyzer and a 532 nm laser, respectively.

## 3. Results and Discussion

**Figure 1:**
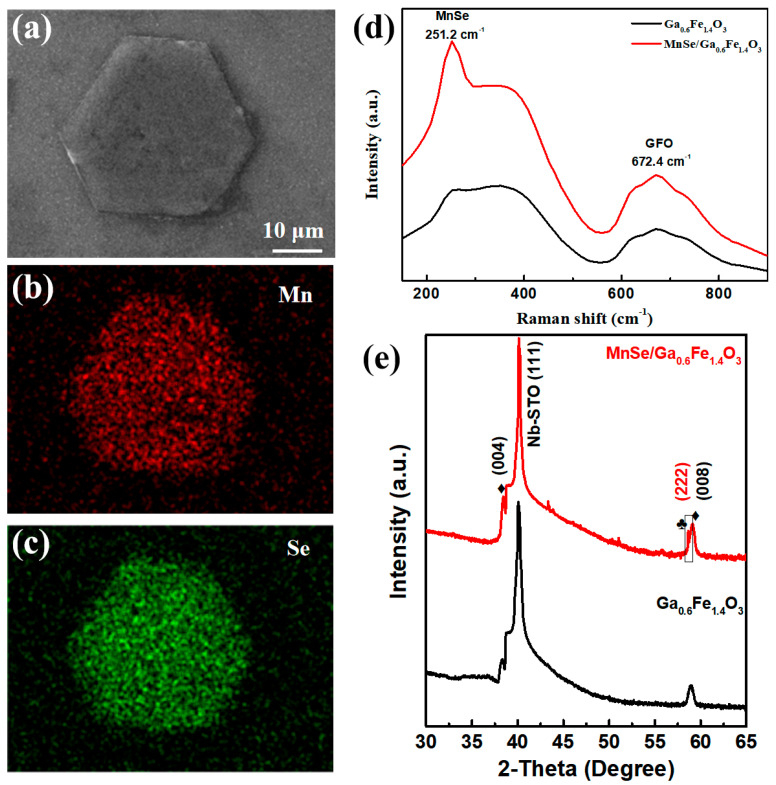
(**a**) SEM diagram of the MnSe/GFO heterostructures. (**b**,**c**) Elemental distribution of Mn and Se in the MnSe/GFO heterostructure. (**d**) Raman spectra comparing the MnSe/GFO heterostructure with a control single GFO film. (**e**) XRD patterns for the synthesized MnSe/GFO heterostructures and a control single GFO film. In the patterns, the ♣ symbol denotes GFO peaks, while the ♦ symbol indicates MnSe peaks.

The surface morphologies of the synthesized MnSe/GFO heterostructures were examined using SEM imaging. The distinct hexagonal shape of MnSe nanosheets is evident in Figure 1a. Figure 1b,c present the EDS elemental mapping of the hexagonal MnSe nanosheet, confirming the uniform distribution of Mn and Se. Additionally, Figure S1 shows the presence of Fe and Ga within the analyzed area. Figure S1d offers quantitative analysis from EDS data, indicating a 1:1 atomic ratio of Mn to Se in the hexagonal MnSe nanosheet, which aligns with the expected stoichiometry.

Furthermore, the structural, magnetic, and optoelectronic characteristics of the MnSe/GFO sample were investigated. Raman spectroscopy provides insights into lattice vibration modes and phase structures. Figure 1d displays the Raman spectral data of a single GFO film and the MnSe/GFO heterostructure, excited via a 532 nm laser. Within the range of 150–890 cm^−1^, the MnSe nanosheet’s signal is observed at 251.2 cm^−1^ [24]. Raman peaks in the GFO film were observed at 351.7 and 672.4 cm^−1^ [25].

To elucidate the crystalline structures of the MnSe/GFO heterostructures, XRD patterns are presented in Figure 1e. The prominent diffraction peaks at 38.3° and 58.9° correspond to the (004) and (006) planes of the GFO films on STO substrates. Additionally, the XRD peak at 58.5° (indicated by ♣) is evident in the MnSe/GFO heterostructure pattern, suggesting that non-layered MnSe nanosheets achieve high stability on the GFO film [26]. XRD data show that the repositioned MnSe nanosheet aligns well with the (111) plane on the GFO film. The lack of peaks for original precursors and impurities confirms the excellent quality of the MnSe/GFO heterostructures. The remaining peaks are ascribed to the (111)-oriented STO substrates.

**Figure 2:**
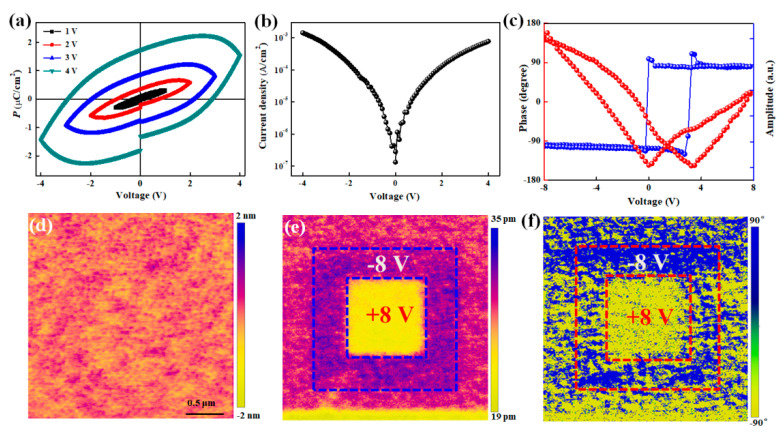
(**a**) Ferroelectric hysteresis loops measured under diverse maximum voltages at a frequency of 1k Hz. (**b**) Leakage current at room temperature as a function of applied bias voltage at 4 V. (**c**) Phase and amplitude of the PFM signals’ dependence on the bias voltage. (**d**–**f**) Surface morphology, corresponding PFM amplitude, and out-of-plane PFM phase images of MnSe/GFO heterostructure.

In order to investigate multiferroic properties in the MnSe/GFO heterostructure, the macroscopic and local ferroelectric properties were systematically characterized in Figure 2. Meanwhile, the macroscopic and local ferroelectric properties also are measured in heterostructures consisting of MnSe nanosheets and 60 nm GFO, as shown in Figure S2. The conductive Nb-STO substrate serves as the bottom electrode. In Figure 2a, the polarization-electric field (P-E) loops of MnSe transferred onto 45 nm GFO are presented. They were assessed at various maximum test voltages, operating at 1 kHz. Notably, regardless of the voltage applied, all loops exhibited an unsaturated behavior, attributed to significant leakage current in the GFO. Figure 2b illustrates the corresponding dependence of current density on voltage bias, depicting the leakage factor. Notably, when applying a voltage of 4 V, the leakage current density can reach 2 × 10^−3^ A/cm^2^. The leakage current in GFO-based heterostructure is a common problem, as previous results show. To weaken the influence of the leakage current effect, the piezo-response force microscopy (PFM) measurement is a useful method to prove the microscopic ferroelectric in MnSe/GFO heterostructure. In Figure 2c, both the phase hysteresis loop and amplitude butterfly curve distinctly demonstrate symmetric ferroelectric switching behavior, underscoring the stability of polarization states within our heterostructure sample. Notably, phase reversal exists at a coercive voltage of <2 V in the tip-polarized position, where the 180° phase contrast obviously indicates polarization switching and thus confirms room-temperature ferroelectricity in this heterostructure.

In Figure 2d, the surface morphology of the GFO layer within the heterostructure is depicted, with a measured roughness of 0.893 nm. Figure 2e,f present PFM amplitude and phase images, respectively, following the creation of box-in-box patterns using a reversed direct current bias of ±8 V. The clear reversals of amplitude and phase contrast demonstrate the polarization switching occurring within the MnSe/GFO heterostructure.

**Figure 3:**
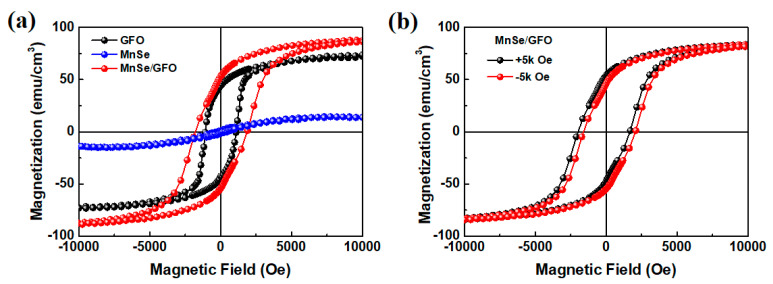
(**a**) Magnetic hysteresis loops obtained from the substrate GFO films, MnSe nanosheets, and MnSe/GFO heterostructures at 10 K under an in-plane magnetic field. (**b**) Magnetic hysteresis loop of the MnSe/GFO heterostructures at 10 K following positive- and negative-field cooling (+5 and −5 kOe, respectively).

To explore the magnetic exchange bias phenomenon in the MnSe/GFO heterostructures, magnetic measurements are discussed in this section. As previously reported, non-layered α-MnSe nanosheets exhibit predominant antiferromagnetism along with minor ferromagnetism [27], while GFO films display room-temperature ferromagnetism [21]. Figure 3a illustrates the magnetic hysteresis loop of an individual MnSe nanosheet, a 45 nm GFO film, and the MnSe/GFO heterostructures at 10 K, with the magnetic field applied in the in-plane direction. Additional data showing the hysteresis loops for GFO films of varying thicknesses are provided in Figure S3. Notably, the MnSe/GFO heterostructure demonstrates higher saturated magnetization and coercive field values compared to the individual materials. This observation indicates a significant magnetic exchange bias effect occurring between the antiferromagnetic component of MnSe and the ferromagnetic GFO.

Moreover, the magnetic hysteresis loop of the MnSe/GFO heterostructure was recorded after cooling the sample from room temperature in the presence of in-plane magnetic fields of ±5 kOe. It is evident that the direction of the horizontal loop shift is opposite to the cooling field for all samples, signifying the existence of the exchange bias effect (EBE) in this heterostructure. The exchange bias field (H_EB_) is calculated as H_EB_ = |H_+_ + H_−_|/2, and the coercive field (H_C_) is determined as H_C_ = |H_+_ − H_−_|/2, where H_+_ and H_−_ denote the right and left coercivity, respectively [28]. As shown in Figure 3b, a pronounced negative coercive field of −1994 Oe and a positive coercive field of 1650 Oe were observed. Consequently, an exchange bias field of 172 Oe and a coercive field of 1822 Oe were obtained in the +5 kOe field-cooling loop. Conversely, when cooled in a −5 kOe field, the center of the magnetic loop shifted towards the positive fields, indicating that the magnetic exchange bias impact is an inherent characteristic of MnSe/GFO heterostructures.

**Figure 4:**
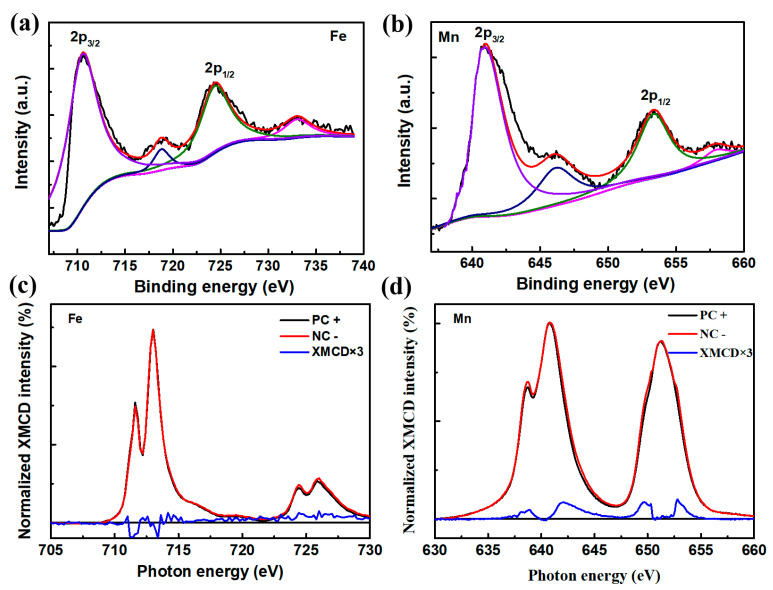
(**a**,**b**) XPS signals from Fe 2*p* and Mn 2*p* orbitals, respectively. (**c**,**d**) The XAS and XMCD of Fe and Mn elements for MnSe/GFO heterostructures at 10 K.

To probe the atomic compositions and magnetic exchange bias impact in the MnSe/GFO heterostructures, XPS and synchrotron X-ray techniques were utilized to examine the states of the core levels. As depicted in Figure 4a, the Fe 2*p* spectra from the MnSe/GFO heterostructure closely resemble those reported for the single GFO films, confirming a predominantly valence state of +3 for the Fe ions. The peaks observed at binding energies of 724.4 and 710.6 eV are due to the spin-orbital coupling forming Fe 2*p*_1/2_ and 2*p*_3/2_, respectively. Additionally, these peaks in the MnSe/GFO exhibited shoulder peaks at 718.8 and 733.0 eV, indicating some surface oxidation [29]. The Mn 2*p* spectra of the heterostructure in Figure 4b show two 2*p* spin orbital coupling signals of Mn 2*p*_3/2_ and 2*p*_1/2_ at 640.9 and 653.3 eV, respectively. Furthermore, the additional peaks at higher binding energies of 646.2 and 658.0 eV suggest minor surface oxidation [30]. This is consistent with the previous single MnSe nanosheet. Additionally, the XPS spectral data of Se and Ga ions were displayed in Figure S4.

To substantiate the magnetic exchange bias impact in the MnSe/GFO heterostructures, synchrotron X-ray measurements of Mn and Fe elements were conducted at 10 K. Polarized photons were incident at a 30° grazing angle onto the MnSe/GFO specimen under an applied field of 5 kOe in fluorescence yield mode [31]. Figure 4c displays the XMCD and XAS spectral data for the Fe L-edge. These spectra show a pre-peak shoulder and an L_3_ main peak of the Fe ion at 711.6 and 712.9 eV, respectively. The shoulder peak corresponds to octahedral sublattice sites within the GFO thin films, while the L_3_ signal arises from the tetrahedral sublattice. In the XMCD spectra, two distinct negative peaks near 711.3 and 713.4 eV confirm the contribution of octahedral Fe^3+^ ions to the macroscopic magnetism. Figure 4d presents the XAS/XMCD measurements at the Mn L-edges. XMCD curves were derived from the difference in XAS signals in negative circle (NC^−^) and positive circle (PC+) orientations relative to the applied field. For enhanced visibility, the XMCD curves have been magnified threefold. The XMCD signal from the Fe *L*_3_ edge indicates that the Fe is aligned parallel to the magnetic field. The obvious XMCD signal from the Mn *L*_3_ edge suggests a small net magnetization exhibited in the MnSe layer and this signal is oriented antiparallel to the Fe. The alignment of Mn antiparallel to the magnetic field incurs a Zeeman energy cost, implying that Mn and Fe must have a compensating antiparallel coupling [32]. This is powerful evidence for the existence of magnetic exchange bias effect in MnSe/GFO heterostructure.

**Figure 5:**
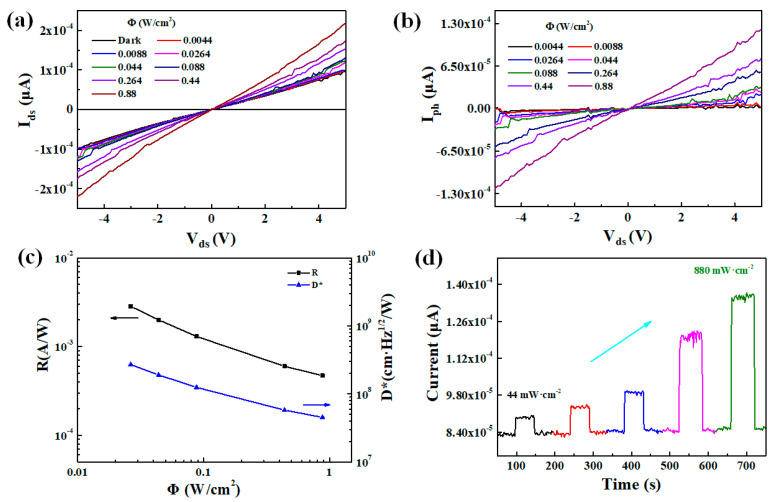
(**a**) Output characteristic curves of the MnSe/GFO photodetector under darkness and different illuminated power density. (**b**) The photocurrent (*I*_ph_) in relation to incident power density. (**c**) The detectivity and responsivity of MnSe/GFO photodetectors as a function of laser power. (**d**) The photoresponse time dependence of light intensity curves of MnSe/GFO at various intensities at 5 V.

Given the prior discussions, it is anticipated that the p-type semiconductive MnSe nanosheet would exhibit excellent optoelectronic properties [33]. To verify if these properties are retained in the MnSe/GFO heterostructures, its optoelectronic and electronic behaviors were investigated. MnSe/GFO photodetectors were constructed through a single-step laser direct writing process, with Cr/Au used for electrode deposition. The photoresponse characteristics were comprehensively assessed under ambient conditions (excitation source = 532 nm laser; α-MnSe optical bandgap = 2.0 eV). Figure 5a demonstrates the output curve obtained under both illuminated and non-illuminated conditions. The source-drain current demonstrates a linear dependence on power density, suggesting an ohmic contact between the electrodes and the MnSe/GFO channels. Figure 5b demonstrates the relationship between the photocurrent (*I*_ph_) and voltage under different incident power densities. *I*_ph_ is calculated as *I*_ph_ = *I*_light_ − *I*_dark_ the difference between the irradiated current, *I*_light_, and the dark current, *I*_dark_). The linear correlation between the *I*_ph_ and the power densities suggests that the photocurrent is primarily determined by absorbed photons. Furthermore, stability and reproducibility under periodic illumination with different incident wavelengths were measured. Figure 5c presents the calculated responsivity (*R*_*λ*_) and detectivity (D*) under variable incident light conditions, indicating the photon-to-electron conversion efficiency and responsiveness of the photodetector. The values of *I*_ph_, D*, and *R*_*λ*_ are crucial metrics for assessing photodetector performance. Utilizing the formulas D* = *R*_*λ*_*S*^1/2^/(2e*I*_dark_)^1/2^ and *R*_*λ*_ = *I*_ph_/*PS* (where *P*, *λ*, and *S* denote the incident laser power density, wavelength, and efficient detection region, respectively), the MnSe device shows outstanding photoresponse to a 532 nm laser at 44 mW/cm^2^, yielding an *I*_ph_ of 2.1 × 10^−5^ μA with a maximum *R*_*λ*_ of 2.8 × 10^−3^ A/W, and a D* of 6.3 × 10^8^ Jones. Figure 5d depicts a monotonically increasing photocurrent with the increase in the light intensity at 532 nm. Consequently, the responsivity in the MnSe/GFO device surpasses those reported for semiconductor materials such as α-MnSe, MoS_2_, and WS_2_ [34,35,36].

## 4. Conclusions

In this study, multiferroic GFO films of varying thicknesses, specifically 45 and 60 nm, were grown on (111)-oriented Nb-STO substrate. Non-layered α-MnSe nanosheet was first transferred onto PDMS through a wedging transfer approach, followed by a mechanical transfer onto the GFO films via a transfer platform. The superior crystalline performance of MnSe/GFO heterostructures was confirmed through SEM, Raman spectroscopy, and XRD analyses. Both macroscopic and local ferroelectric properties were comprehensively assessed using polarization-electric field loops and PFM technology. The leakage current density reached as high as 2 × 10^−3^ A/cm^2^ under a 4 V bias, with a remanent polarization of approximately 1.5 μC/cm^2^. Moreover, PFM amplitude and phase scans after patterning box-in-box structures further corroborated the ferroelectric characteristics. A significant enhancement in saturated magnetization and coercive field, along with antiparallel signals of Mn and Fe ions observed through synchrotron X-ray analysis, demonstrated the presence of a magnetic exchange bias interaction within the MnSe/GFO heterostructures. Additionally, the heterostructure demonstrated exceptional photodetection performance, with a detectivity of 6.3 × 10^8^ Jones and a photoresponsivity of 2.8 × 10^−3^ A/W at a luminescence wavelength of 532 nm. Thus, the multifunctional attributes of this heterostructure, encompassing multiferroicity, magnetic coupling, and photodetection, hold great promise for the development of next-generation devices.

## Data Availability

Data will be made available on request.

## Supplementary Materials

The following supporting information can be downloaded at: https://www.mdpi.com/article/10.3390/ma18030586/s1, Figure S1: SEM image of MnSe/GFO heterostructure; Figure S2: Ferroelectric hysteresis measurements; Figure S3: The magnetic hysteresis loops of different GFO films; Figure S4: The XPS results.
