# Supplementary material for: The Multiferroic, Magnetic Exchange Bias Effect, and Photodetection Multifunction Characteristics in MnSe/Ga0.6Fe1.4O3 Heterostructure
Source: Materials (Basel). 2025 Jan 27;18(3):586. doi: 10.3390/ma18030586 (PMC11818127; doi:10.3390/ma18030586)
Supplement: Supplementary file 1 [file materials-18-00586-s001.zip › materials-3384355-supplementary.pdf]

Supporting Information

The multiferroic, magnetic exchange bias effect, and photodetection multifunction characteristics in MnSe/$Ga_{0.6}Fe_{1.4}O_3$ heterostructure

Ye Zhao[1†], Ruilong Yang[1,2 †], Ke Yang[1], Jiarui Dou[1], Jinzhong Guo[1], Xiaoting Yang[1], Guowei Zhou[1,2,*], and Xiaohong Xu[1,2,*]

[1] School of Chemistry and Materials Science of Shanxi Normal University & Key Laboratory of Magnetic Molecules and Magnetic Information Materials of Ministry of Education, Taiyuan 030006, China

[2] Research Institute of Materials Science of Shanxi Normal University, Taiyuan 030006, China

[†] These authors contributed equally to this paper.

[*] Corresponding author (email: zhougw@sxnu.edu.cn; xuxh@sxnu.edu.cn)

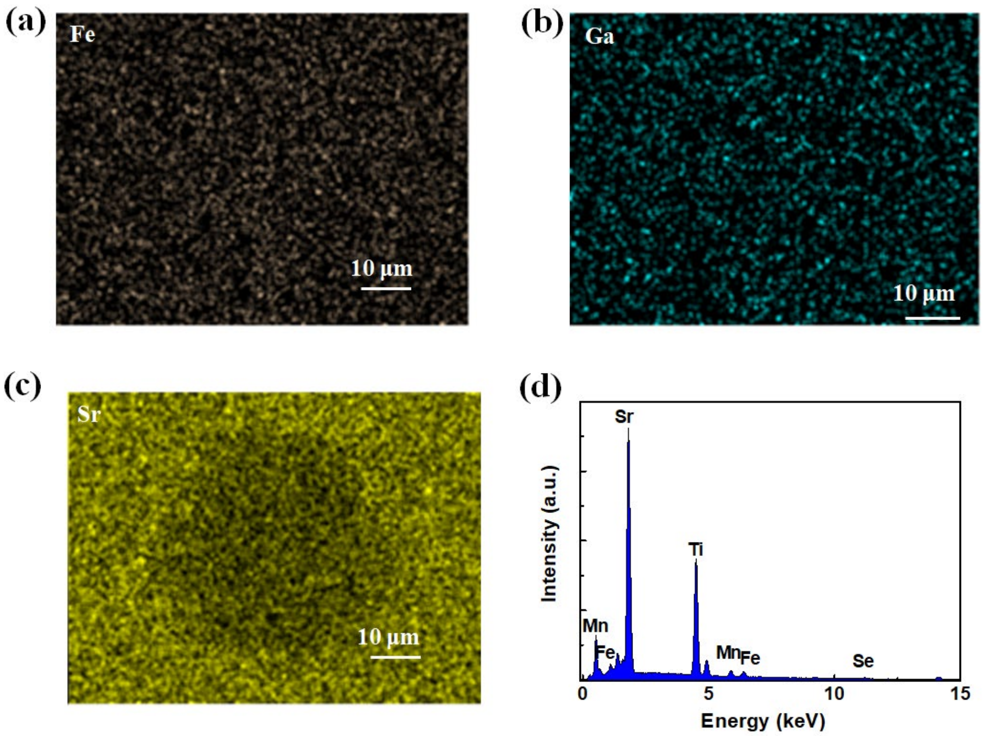

Figure S1 SEM image of MnSe/GFO heterostructure. (a-b) The Fe and Ga elemental images of MnSe/GFO heterostructure. The hexagonal shape of MnSe nanosheet is disappeared due to the large percentage of GFO. (c) The Sr elemental image of STO substrates. (d) The SEM-EDS analysis of MnSe/GFO heterostructure on grids.

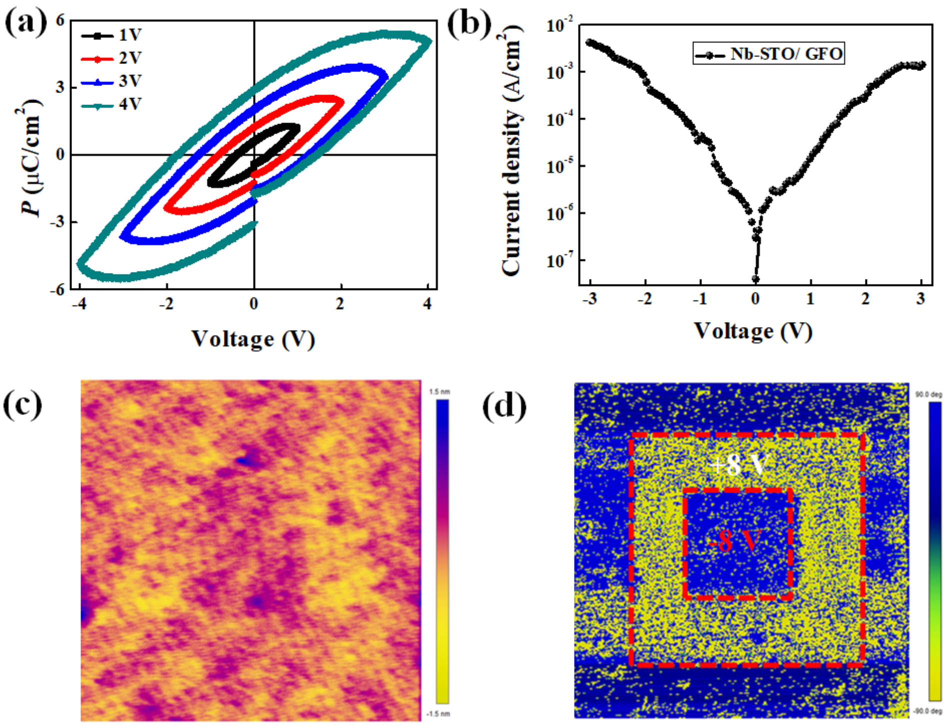

Figure S2 (a) Ferroelectric hysteresis loops of MnSe/GFO heterostructure measured under 1 to 4 voltages at a frequency of 1k Hz. The thickness of GFO is 60 nm in this heterostructure. (b) Leakage current at room temperature as a function of applied bias voltage as 3 V. (c-d) Surface morphology, corresponding out-of-plane PFM phase images of MnSe/GFO heterostructure. The PFM images were first scanned by -8 V DC voltage in an outer box of 2×2 μm^2 followed by +8 V DC voltage in an inner box of 1×1 μm^2.

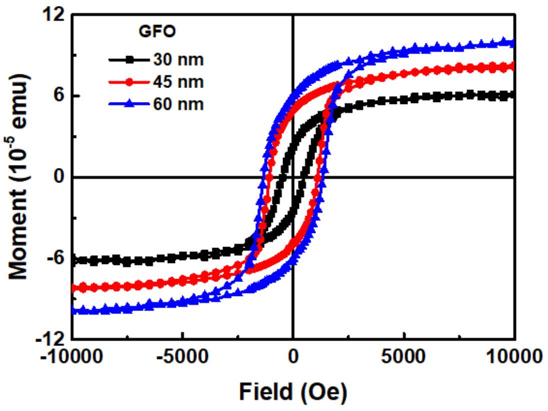

Figure S3 The magnetic hysteresis loops of different thickness GFO films (30-60 nm) along in-plane magnetic field at 10 K, respectively.

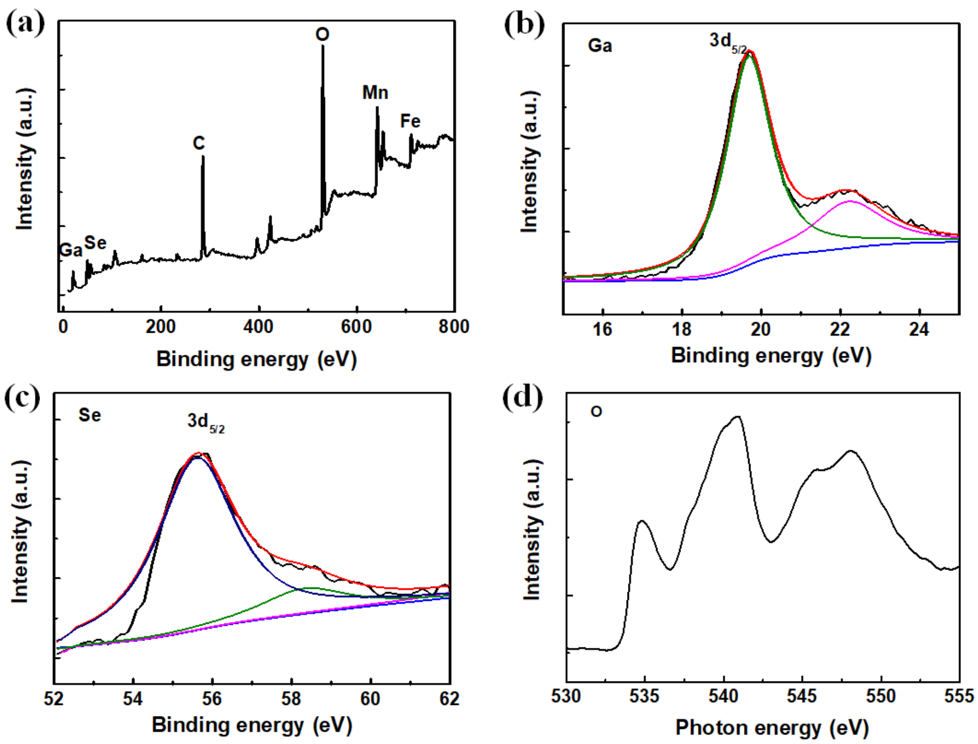

Figure S4 (a) The full spectrum diagram of MnSe/GFO heterostructure, where the Ga, Fe, Mn, Se elements can be observed. (b-c) The XPS fine spectra of Ga 3*d* and Se 3*d* orbitals, respectively. (d) The X-ray absorption spectroscopy of O element for MnSe/GFO heterostructure.
